# Increasing the accuracy of proteomic typing by decellularisation of amyloid tissue biopsies

**DOI:** 10.1016/j.jprot.2017.06.016

**Published:** 2017-08-08

**Authors:** P. Patrizia Mangione, Giuseppe Mazza, Janet A. Gilbertson, Nigel B. Rendell, Diana Canetti, Sofia Giorgetti, Luca Frenguelli, Marco Curti, Tamer Rezk, Sara Raimondi, Mark B. Pepys, Philip N. Hawkins, Julian D. Gillmore, Graham W. Taylor, Massimo Pinzani, Vittorio Bellotti

**Affiliations:** a Wolfson Drug Discovery Unit, Centre for Amyloidosis and Acute Phase Proteins, University College London, London, UK; b Department of Molecular Medicine, Institute of Biochemistry, University of Pavia, Pavia, Italy; c Institute for Liver and Digestive Health, University College London, London, UK; d National Amyloidosis Centre, University College London and Royal Free Hospital, London, UK; e CEINGE and Department of Chemical Sciences, University of Naples, Naples, Italy

**Keywords:** Amyloidosis, Decellularisation, Proteomics, Amyloid typing

## Abstract

Diagnosis and treatment of systemic amyloidosis depend on accurate identification of the specific amyloid fibril protein forming the tissue deposits. Confirmation of monoclonal immunoglobulin light chain amyloidosis (AL), requiring cytotoxic chemotherapy, and avoidance of such treatment in non-AL amyloidosis, are particularly important. Proteomic analysis characterises amyloid proteins directly. It complements immunohistochemical staining of amyloid to identify fibril proteins and gene sequencing to identify mutations in the fibril precursors. However, proteomics sometimes detects more than one potentially amyloidogenic protein, especially immunoglobulins and transthyretin which are abundant plasma proteins. Ambiguous results are most challenging in the elderly as both AL and transthyretin (ATTR) amyloidosis are usually present in this group. We have lately described a procedure for tissue decellularisation which retains the structure, integrity and composition of amyloid but removes proteins that are not integrated within the deposits. Here we show that use of this procedure before proteomic analysis eliminates ambiguity and improves diagnostic accuracy.

**Significance:**

Unequivocal identification of the protein causing amyloidosis disease is crucial for correct diagnosis and treatment. As a proof of principle, we selected a number of cardiac and fat tissue biopsies from patients with various types of amyloidosis and show that a classical procedure of decellularisation enhances the specificity of the identification of the culprit protein reducing ambiguity and the risk of misdiagnosis.

## Highlights

• Clinical management of systemic amyloidosis depends on the fibrillar protein.
• Decellularisation of tissue biopsies preserves amyloid.
• Decellularisation of biopsies enhances specificity in MS-based proteomic diagnosis.
• This procedure eliminates ambiguity between two potential amyloidogenic proteins.

## Graphical abstract

**Figure:**
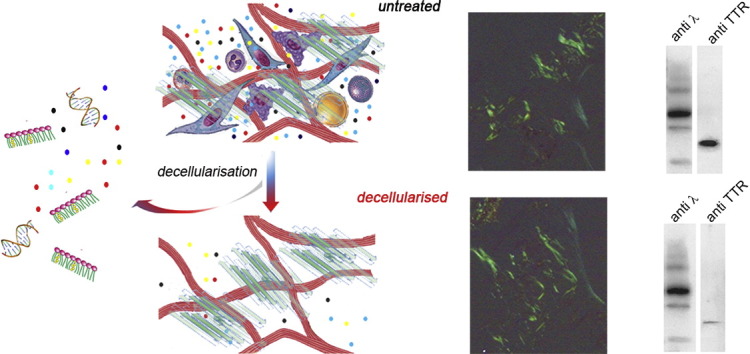

## 1 Introduction

One of the major therapeutic strategies of systemic amyloidosis consists in reducing the production and thus the abundance of the protein which aggregates to form extracellular amyloid deposits. Therapy is therefore dictated by amyloid fibril typing in each case [1]. Most crucially, the very toxic and expensive cytotoxic chemotherapy essential in AL amyloidosis is not beneficial in other forms of amyloidosis and can be extremely dangerous [2]. Proteomic analysis of amyloid deposits provides direct chemical characterization of the proteins present and is complementary to immunohistochemical staining for known amyloid fibril proteins and gene sequencing to identify known or potential amyloidogenic mutations. It has been reported that proteomics unambiguously identifies the major amyloid constituent in the large majority of cases [3], but in some cases more than one potentially amyloidogenic protein, even though with different score, is detected in the amyloid specimen and for these patients an accurate diagnosis can become quite challenging [4], [5]. Most commonly the ambiguity can be caused by the co-presence in the specimen of both immunoglobulin light chains and transthyretin, both of which are abundant in the plasma and cause the two most common forms of systemic amyloidosis. Furthermore, the prevalence of wild-type ATTR amyloidosis, uniquely a disease of the elderly, has recently been recognised and monoclonal gammopathy with increased production of monoclonal immunoglobulin light chains, the precursor of AL amyloid fibrils, is common in this age group [6]. Indeed, up to 20% of patients with ATTR amyloidosis have an incidental monoclonal protein in serum or urine [7].

An additional technical problem arises from the increasing and highly recommended use of fine needle fat aspiration biopsies [8] for diagnosis and typing of amyloid. Proteomic analysis of standard excision or resection biopsies involves laser capture microdissection of the amyloid deposits themselves, selectively concentrating the fibrils, but this step is omitted with fine needle fat aspiration samples. Contamination by abundant plasma proteins, not necessarily related to amyloid formation *in vivo*, is common in these specimens.

We have lately demonstrated that application of tissue decellularisation to amyloidotic organs and tissues leaves extracellular matrix proteins and amyloid deposits intact [9]. Here we show that subjecting biopsies to a rapid tissue decellularisation protocol before digestion and mass spectrometry (MS) analysis, eliminates background contamination and enables unequivocal identification of the actual amyloid fibril protein.

## 2 Methods

### 2.1 Patients

Patients attending the National Amyloidosis Centre in the Centre for Amyloidosis and Acute Phase Proteins at UCL, London, UK, all provided informed written consent in accordance with the Declaration of Helsinki. Endomyocardial biopsies were received from 3 patients. Patient 1: a 73 year old man with κ myeloma presented with periorbital bruising, macroglossia and heart failure with amyloid cardiomyopathy. ^99m^Tc-3,3-diphosphono-1,2-propanodicarboxylic acid (^99m^Tc-DPD) scan showed Perugini grade 3 cardiac uptake but no visceral amyloid deposits were detected by serum amyloid P component (SAP) scintigraphy. No mutations were detected on sequencing of his *TTR* and *APOA1* genes. His myeloma was treated with cyclophosphamide vincristine dexamethasone (CVD) chemotherapy. Patient 2: a 65-year-old man with heart failure and amyloid cardiomyopathy but no abnormality on Tc–DPD or SAP scintigraphy, no monoclonal protein in serum or urine and wild-type *TTR* and *APOA1* genes. Patient 3: a 67-year-old man with chronic lymphocytic leukaemia and a monoclonal κ paraprotein who presented with heart failure, amyloid cardiomyopathy and autonomic neuropathy. TC-DPD scan showed Perugini grade 2 uptake but no visceral amyloid deposits were detected by SAP scintigraphy. *TTR* gene sequencing showed that he was heterozygous for the known amyloidogenic mutation encoding T60A TTR. Fat biopsies were received from 7 other patients in which more than one potentially amyloidogenic protein was detected in the untreated tissue specimen.

### 2.2 Laser capture microdissection and proteomic mass spectrometry analysis

Sections (6 μm) of formalin-fixed paraffin-embedded tissue on Director™ slides, stained with alkaline alcoholic Congo red (AMRESCO, Solon, OH, USA) [10] and haematoxylin (Pioneer Research Chemicals, Colchester, UK), were viewed under intense cross polarized light. Areas showing the pathognomonic apple green birefringence of amyloid were excised by laser capture microdissection and trapped on adhesive caps of microcentrifuge tubes. Following the method of Rodriguez et al. [11], proteins were extracted from each sample into 10 mM Tris/1 mM EDTA/0.002% Zwittergent buffer solution (35 μl) by heating (99 °C, 1.5 h) followed by sonication (1 h) and then digested with trypsin (1.5 mg *w*/*v*) overnight (~ 18 h) at 37 °C. Each digested sample was reduced with dithiothreitol (50 μg) at 99 °C for 5 min, freeze dried, reconstituted in 0.1% v/v trifluoroacetic acid in HPLC grade water (20 μl) and analysed by HPLC-MSMS.

Tryptic digests were applied to a trap column (180 μm ID × 20 mm bed, 5 μm Symmetry C18 packing; Waters Corporation, Massachusetts, USA) and separated on a reverse phase column (100 μm ID × 150 mm bed, 5 μm C18 packing; Nikkyo Technos Company Ltd., Tokyo, Japan) using a linear gradient from 1% to 60% acetonitrile/water over 44 min at 400 nl/min. Ten partial system washes (injector and trap) using injection boluses containing formic acid, ammonia, methanol and acetonitrile followed by a full solvent blank were run after each sample. Nanoflow liquid chromatography-electrospray tandem MS was performed using a Waters nanoACQUITY™ UPLC system (Waters Ltd., Elstree, Hertfordshire, UK) coupled to a Thermo Scientific Orbitrap Velos Mass Spectrometer (Thermo Electron, Bremen, Germany) operated in the positive ion mode. Each tryptic digest was analysed in three technical replicates unless otherwise stated (Supplementary Table1).

Instrument control and data acquisition used Thermo Scientific Xcalibur Version 2.1. MS data files were analysed using MASCOT software (Matrix Science, London, UK) [12] to search the SwissProt database. Searches were conducted with trypsin as the digestion enzyme (with 2 missed cleavages) and oxidation of methionine set as a variable modification; mass tolerances were 10 ppm for precursor ions, 0.60 Da for fragment ions with possible charge states of + 2, + 3 and + 4. Protein identities were expressed in terms of MASCOT probability-based scores with a significance value set at *p* < 0.05 (http://www.matrixscience.com/help/scoring_help.html).

In addition Mascot output data files were also analysed and validated by the use of Scaffold 4.6.1 (Proteome Software, Inc., Portland, OR). This tool uses a Local False Discovery Rate (LFDR)-based scoring system for peptide validation based on a Bayesian approach to confirm peptide probabilities. The likelihood of peptides is calculated on parent ion mass accuracy and parent ion delta masses [13], [14]. Filtering parameters for protein identification by Scaffold were set at a protein threshold confidence level > 99%, a minimum of 2 assigned peptides with a probability > 95%.

In addition, to increase confidence in protein identification and peptide validation, Scaffold software was run using Mascot and X!Tandem search engines.

A semi-quantitative analysis was performed on the group of fat aspirates by considering the total number of spectra matched to a single protein group (TS) [15]; TS values in the three replicates per sample were averaged. Ratios of the resulting TS between non-amyloid and amyloid protein in both untreated and decellularised samples were calculated and plotted. In addition, we carried out a label free quantification (LFQ) of proteins using MaxQuant software (version 1.5.8.3), which is based on the ion intensities of the extracted ion chromatogram [16].

For the first and the main search, peptide mass tolerances were 20 ppm and 4.5 ppm respectively, whereas for MS/MS the threshold was 20 ppm. For the protein identification, a minimum of 2 peptides and at least 1 unique peptide were set. The search was performed in revert decoy mode with peptide spectra matches false discovery rate (PSM FDR), protein FDR and site decoy fraction set at 0.01. An average of LFQ of 3 replicates for each experiment was determined and the ratio between LFQ of non-amyloid and amyloid protein in untreated and decellularised samples was calculated and plotted.

Statistical analysis for the group of 7 fat biopsies was performed by using the Mann Whitney test on GraphPad Prism 5 software (GraphPad Prism Inc., San Diego, CA). Statistically significant differences between the untreated and decellularised TS (or LFQ) ratios are indicated by *p* < 0.05.

### 2.3 Decellularisation protocol

Fresh, unfixed, snap frozen cardiac or adipose tissue biopsies were decellularised by sequential washing with 500 mM NaCl containing 2 mM calcium chloride; 4% w/v sodium deoxycholate; and Tris-HCl containing 140 mM NaCl, 2 mM calcium chloride, pH 8.0 (all reagents from Sigma-Aldrich, St. Louis, MO, USA). All the steps were carried out at room temperature using a minimal volume of the respective buffer in Eppendorf safe-lock tubes placed in a tissuelyser (Qiagen, Hilden, Germany) at 25 Hz per 2 min. Typically two set of washings were repeated for the tissue to become translucent. Decellularised cardiac biopsy specimens were then formalin-fixed, wax-embedded for Congo red staining [10], laser dissection and proteomic MS analysis. In addition fresh untreated and decellularised fat biopsies were digested with trypsin (Promega, Madison, WI, USA) without prior fixation or any previous processing and then analysed by liquid chromatography mass spectrometry mass spectrometry (LCMSMS) as above.

At this stage, the decellularisation does not allow a quantitative measurement of the yield of amyloid at the end of the procedure. Qualitative analysis carried out by microscopic analysis of Congo red stained specimen, however, reveals that the intensity of the green birefringence remains unaltered after the decellularisation.

### 2.4 Immunoblotting

Sections of unfixed cardiac biopsies, both untreated and decellularised, were analysed by immunoblotting. Blotted-dry tissue was ultrasonicated in the presence of Laemmli buffer (~ 10 μg tissue/μl). After heating at 95 °C for 5 min and centrifugation for 10 min at 10,600*g*, reduced denatured supernatants (3 μl) were subjected to SDS-PAGE in precast 15% gradient polyacrylamide gels (Excel, GE Healthcare, Little Chalfont, UK) and then blotted onto an activated PVDF membrane. Polyclonal rabbit anti-κ light chain (4 μg/ml, Dako, Glostrup, Denmark) or sheep anti-human TTR (3 μg/ml, The Binding Site, Birmingham, UK) antibodies were used to identify their cognate antigens and were detected respectively by peroxidase-conjugated polyclonal sheep anti-rabbit (25 × 10^− 3^ μg/ml, Dako, Glostrup, Denmark) or anti-sheep (13 × 10^− 3^ μg/ml) immunoglobulins, followed by 1:10,000 StrepTactin-HRP (Biorad, Hercules, CA, USA) for identification of the molecular weight standards (Precision Plus Protein WesternC, Biorad, Hercules, CA, USA). Peroxidase activity was visualized using a chemiluminescent substrate (Immobilon Western, Millipore, Billerica, MA, USA) and a FluorChem M system (Proteinsimple, San Jose, CA, USA).

## 3 Results and discussion

**Fig. 1:**
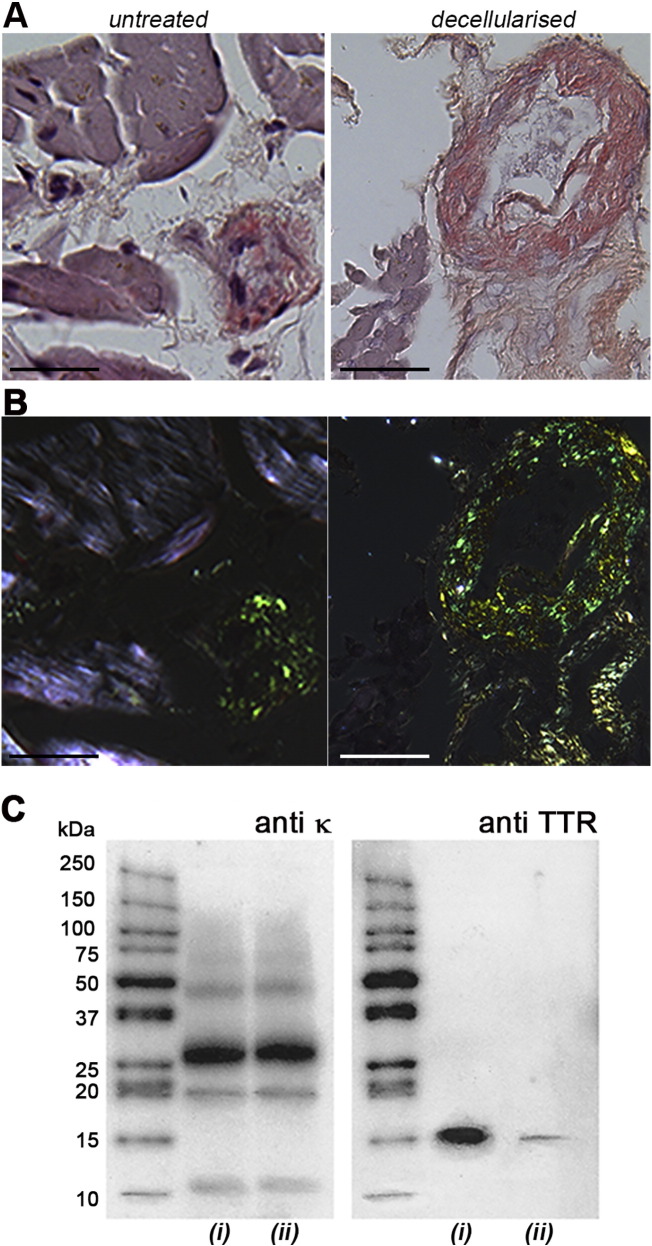
Amyloid in untreated and decellularised endomyocardial biopsy from patient 1. (A) Bright field view showing Congophilic material in Congo red stained [10] sections of formalin-fixed, wax-embedded untreated and decellularised tissue. (B) Intense cross-polarized light view of same sections showing retention of the pathognomonic apple green birefringence of amyloid after decellularisation. Images from Leica DM4000 microscope with Leica DFC7000 T camera. Scale bar, 50 μm. (C) Immunoblot analysis of fresh unfixed untreated (*i*) or decellularised (*ii*) biopsies after reduced SDS homogenous 15% PAGE.

We demonstrated that decellularisation of whole amyloidotic mouse organs fully retained the structure and functional properties of the amyloid deposits in the extracellular space [9]. The procedure involved sequential perfusion with water, detergent and DNase. Here we adapted the procedure to decellularise very small human tissue biopsies in order to improve the specificity of proteomic MS analysis for amyloid typing. Only a limited number of sequential washing steps were carried out using saline buffers and deoxycholate (see methods) with a 1:40 ratio of biopsy weight/buffer volume; the process was extremely rapid and loss of tissue material was minimized. The effect of decellurarisation on even tiny samples is exemplified by the results shown in Fig. 1 on the endomyocardial biopsy (Fig. 1) obtained from a myeloma patient (patient 1) presenting an increased circulating free monoclonal κ light chain concentration and, a high grade cardiac Tc-DPD scan uptake that is a condition typical of ATTR amyloidosis, but rarely seen in cardiac AL amyloidosis [7]. Mascot analysis of mass spectra, after routine proteomic processing of fixed tissue, showed both κ light chain and TTR were present. Mascot output data were then combined and analysed in Scaffold which assigned same probabilities (> 99%) to both κ and TTR (Table 1A, patient 1). This is a typical case in which management and prognosis are radically different if the amyloid protein is TTR or κ light chain. Fresh cardiac biopsy from patient 1 was decellularised and, subsequently, fixed and wax-embedded for Congo-red staining, laser microdissection, trypsin digestion and MS analysis. Bright field imaging after the decellularisation procedure (Fig. 1A) confirmed removal of all the cells without affecting the abundant green birefringent amyloid deposits in the Congo red stained material viewed in cross-polarized light (Fig. 1B). A remarkable change in the results of both immunoblotting and MS analysis was observed. The original blot (Fig. 1C) showed κ light chains with heterogeneous masses, consistent with full-length protein and fragments, and a single TTR band at the mass of full length protomer. The anti-κ immunostaining pattern was unchanged after decellularisation but the intensity of the TTR band was notably reduced. Proteomic analysis of the decellularised sample (Table 1A, patient 1) by Mascot and Scaffold confirmed that κ light chain was the only species detectable after decellularisation.

**Table 1:** Proteomic MS analysis of untreated and decellularised cardiac (A) and fat (B) biopsies.

A										
Cardiac biopsy	Untreated					Decellularised				
Patient	Amyloid score mascot	Scaffold results				Amyloid score mascot	Scaffold results			
		P (%)	EUP	EUS	TS		P (%)	EUP	EUS	TS
1	κ, 184	> 99	4	5	7	κ, 412	> 99	5	6	13
	TTR, 86	> 99	3	3	4	TTR, 0	ND	0	0	0
2	λ, 180	> 99	3	5	7	λ, 222	> 99	6	8	12
	κ, 107	ND	0	0	0	κ, 19	ND	0	0	0
3	TTR, 794	> 99	10	14	21	TTR, 973	> 99	10	13	30


B										
Fat aspirate	Untreated					Decellularised				
Patient	Amyloid score mascot	Scaffold results				Amyloid score mascot	Scaffold results			
		P (%)	EUP	EUS	TS		P (%)	EUP	EUS	TS
4	λ, 755	> 99	11	15	37	λ, 1788	> 99	10	14	64
	κ, 733	> 99	8	12	22	κ, 539	> 99	5	9	16
5	λ, 824	> 99	12	17	40	λ, 1235	> 99	11	17	56
	TTR, 451	> 99	8	10	13	TTR, 348	> 99	7	8	9
6	λ, 221	> 99	5	7	9	λ, 349	> 99	6	8	18
	κ, 39	> 99	1	1	2	κ, 0	ND	0	0	0
7	λ, 301	> 99	5	6	13	λ, 595	> 99	7	10	25
	TTR, 54	> 99	1	1	1	TTR, 76	> 99	1	1	1
8	λ, 465	> 99	4	6	19	λ, 589	> 99	5	7	22
	κ, 252	> 99	4	5	8	κ, 0	ND	0	0	0
9	TTR, 585	> 99	7	8	12	TTR, 1758	> 99	15	25	54
	κ, 609	> 99	5	7	15	κ, 230	> 99	3	3	7
10	TTR, 693	> 99	12	18	23	TTR, 236	> 99	7	9	12
	κ, 257	> 99	4	6	10	κ, 33	> 99	1	1	1

Proteins identified in each sample with Mascot score probabilities and Scaffold software results. P: Protein identification probability; EUP: Exclusive Unique Peptide count (number of unique peptides only associated with this protein); EUS: Exclusive Unique Spectrum count (number of unique spectra only associated with this protein); TS: Total Spectrum count (number of total spectra associated with this protein including those shared with other proteins). ND: not detected. Mean of three technical replicates (see Supplementary Table 1 for the complete list of values) are given for untreated and decellularised cardiac biopsies except for untreated samples for patient 1 and 2 due to lack of material.

Mascot identified both κ and λ light chains in the cardiac biopsy of patient 2 (Table 1A, patient 2) whereas Scaffold detected only λ. After decellularisation, however, the Mascot score for λ light chain increased whereas the κ light chain became undetectable. The Scaffold parameters, after decellularisation, confirmed that λ light chain was the only fibril protein. Although discrimination between κ and ʎ does not affect current treatment for AL amyloidosis, this case shows that the procedure allows unequivocal identification of the fibril protein.

Although in patient 3 we found only TTR in untreated biopsy and the amyloid typing was certain, we checked the protein composition after decellularisation. Similar, or even better, scores for TTR were obtained after treatment (Table 1A), thus confirming that fibrillar TTR is not removed by this procedure. There was sufficient material available from this patient to estimate the variability of the procedure with three separate technical replicates of both control and decellularised tissue. For the first two patients three replicates could be carried out only for the decellularised samples due to lack of material of the untreated samples. In each case, decellularisation did not alter the identification of the amyloid component (Supplementary Table 1) and retain at least two of the so called signatures proteins for amyloid, serum amyloid P component (SAP), apolipoprotein E, apolipoprotein A-IV [17], together with vitronectin and clusterin [5] (Supplementary Table 2).

The decellularisation approach is clearly suitable for examining unfixed frozen human tissue, however it would be unusual to obtain such material in a normal clinical situation. In contrast, fresh fat aspirates are routinely collected in our clinic, and so we investigated whether the same simple decellularisation procedure could be applied to aid proteomics analysis of fats.

**Fig. 2:**
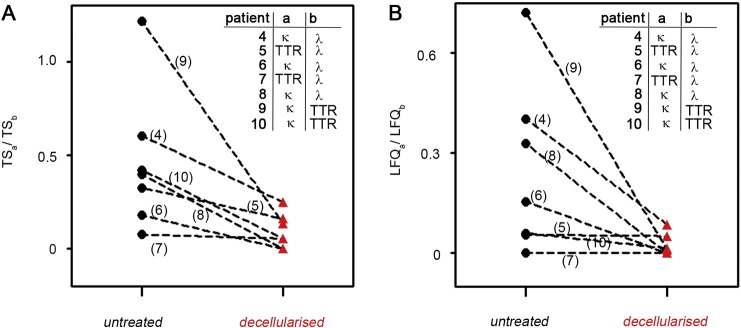
Improving specificity after decellularisation of fat aspirates. (A) In the presence of two potential amyloidosis-associated proteins identified by Mascot and Scaffold, ratios between soluble (a) and fibrillar protein (b) were calculated using their corresponding total spectra count (TS) (see Table 1B) before (black circles) and after decellularisation (red triangles) of fat aspirates. (B) Alternatively ion intensities of the extracted identified peaks (LFQ) of the two potential culprit proteins were quantified using MaxQuant software (Supplementary Table 3). Ratios of the averaged LFQ values for the 3 replicates per sample were plotted as above. a: soluble protein; b: fibrillar protein; black circles: untreated; red triangles: decellularised.

The effect of decellularisation on fat aspirates is summarized in Table 1B and Supplementary Table 1. A semi-quantitative analysis was carried out by considering the Scaffold total spectra count ratio between soluble versus fibrillar protein (Fig. 2A). In three cases (patients 4, 6 and 8), the decellularisation improved the discrimination between k and ʎ light chains as major constituent of the amyloid deposits. In other cases, in which light chains and TTR were both detectable in the tissue specimens (patients 5, 7, 9 and 10), decellularisation increased specificity of amyloid typing as reflected in the score of the fibrillar protein and the ratio between the total peptides for the soluble and fibrillar proteins respectively.

In one case (patient 10), due to partial loss of tissue material, after decellularisation the proteomic scores for both TTR and light chains diminished, but nevertheless the ratio of the respective peptides gives clear indication on the fibrillar constituent. Improvement in the specificity of amyloid typing was overall confirmed when we considered the area of the identified peaks (LFQ values by MaxQuant software in Supplementary Table 3) rather than the total spectra count (Fig. 2B). Impact of decellularisation was significant (*p* < 0.05) on both TS and LFQ ratios between soluble versus fibrillar protein.

For all samples the retrospective clinical and pathologic review of the cases was consistent with the proteomic results (Supplementary Table 4).

Peptide spectra representing all the signature proteins for amyloid [5], [17], [18] were present in the samples before and after decellularisation (Supplementary Table 2).

## 4 Conclusion

It is widely accepted that proteomics is becoming an invaluable tool for diagnosis of systemic amyloidosis [17], [19] and represents a technique under rapid evolution in terms of pre-analytical treatment of samples, technological progress of spectrometers, and bioinformatics analysis of raw data. Our study mostly focuses on the possibility to get better results just intervening on the pre-analytical treatment of the tissue specimens.

The comparative analysis of proteomics of amyloid tissue biopsies before and after decellularisation reveals that this is a swift and straightforward procedure which improves the accuracy and unequivocal identification of amyloid fibril type. It is particularly helpful in cases where results of the current routine proteomic procedure and of immunostaining are equivocal. Decellularisation is readily applicable to both cardiac and fat biopsies. Fine needle fat aspiration is safer, simpler, minimally invasive and less expensive than organ biopsy and laser microdissection is not required for the proteomic analysis of the decellularised fat aspirates [17]. Ready application of decellularisation to fat tissue specimen is particularly important as, just recently, fat aspirate analysis was recommended to get an earlier diagnosis of the disease and therefore a better therapeutic response [8]. In conclusion, the increased sensitivity and specificity of amyloid diagnosis enabled by decellularisation of fresh unfixed fat aspirate samples, before they undergo proteomic MS analysis, may represent a valuable procedure for the proteomic characterization of amyloid deposits in tissue biopsies.

Decellularisation can be considered as a chemical microdissection of the amyloid deposits where fibrils, due to their very high stability, do not dissociate and remain resistant to the extraction with buffer containing detergents. Thermodynamic stability studies of prototypic amyloid fibrils [20] suggest that these aggregates are more stable than many other types of reversible protein-protein interactions occurring in tissue. This property clearly represents an advantage for diagnosis but is a major obstacle to the pharmacological removal of amyloid *in vivo*.

## Authorship contributions

The study was conceived, designed and supervised by V.B. P.P.M., G.M., J.A.G., N.R., D.C., S.G., L.F., M.C., T.R., G.W.T. performed research. M.B.P., P.N.H., J.D.G., M.P. contributed to experimental design and discussion. All the authors analysed and interpreted the data. The paper was written by P.P.M., J.D.G., G.W.T., V.B. and M.B.P. reviewed and approved by all co-authors.

## Disclosure of conflicts of interest

The authors declare no competing financial interests.

## Appendix A Supplementary data

Supplementary tables. Image 1

Protein and peptide identification by Mascot. Image 2

Label free quantification by MaxQuant. Image 3
