# Supplementary material for: Increasing the accuracy of proteomic typing by decellularisation of amyloid tissue biopsies
Source: J Proteomics. 2017 Aug 8;165:113–8. doi: 10.1016/j.jprot.2017.06.016 (PMC5571436; doi:10.1016/j.jprot.2017.06.016)
Supplement: Supplementary file 1 — Supplementary tables. [file mmc1.pdf]

Supplementary material

Increasing the accuracy of proteomic typing by decellularisation of amyloid tissue biopsies

P. Patrizia Mangione, Giuseppe Mazza, Janet A. Gilbertson, Nigel B. Rendell, Diana Canetti, Sofia Giorgetti, Luca Frenguelli, Marco Curti, Tamer Rezk, Sara Raimondi, Mark B. Pepys, Philip N. Hawkins, Julian D. Gillmore, Graham W. Taylor, Massimo Pinzani, Vittorio Bellotti.

Supplementary Table 1

Supplementary Table 2

Supplementary Table 3

Supplementary Table 4

Supplementary References

Full datasets with protein and peptide identifications by Mascot (and LFQ intensity by MaxQuant) have been uploaded separately as supplementary files.

Supplementary Table 1. Proteomic MS analysis of untreated and decellularised cardiac and fat biopsies.

Cardiac biopsy	*Untreated*					*Decellularised*				
Patient	***Amyloid Score MASCOT***	***SCAFFOLD RESULTS***				***Amyloid Score MASCOT***	***SCAFFOLD RESULTS***			
		P (%)	***EUP***	***EUS***	***TS***		***P (%)***	***EUP***	***EUS***	***TS***
***1**						κ, 242	>99	6	7	10
	κ, 184	>99	4	6	8	κ, 439	>99	5	6	12
						κ, 554	>99	5	6	16
						TTR, 0	ND	0	0	0
	TTR, 86	>99	3	3	4	TTR, 0	ND	0	0	0
						TTR, 0	ND	0	0	0
***2**						λ, 310	>99	5	7	11
	λ, 180	>99	3	5	7	λ, 87	>99	5	6	7
						λ, 268	>99	6	9	15
						κ, 28	ND	0	0	0
	κ, 107	ND	0	0	0	κ, 0	ND	0	0	0
						κ, 30	ND	0	0	0
**3**	TTR, 836	>99	7	13	17	TTR, 969	>99	9	14	27
	TTR, 700	>99	9	11	18	TTR, 976	>99	10	13	34
	TTR, 846	>99	14	17	29	TTR, 975	>99	10	13	30

Fat aspirate	*Untreated*					*Decellularised*				
Patient	***Amyloid Score MASCOT***	***SCAFFOLD RESULTS***				***Amyloid Score MASCOT***	***SCAFFOLD RESULTS***			
		P (%)	***EUP***	***EUS***	***TS***		***P (%)***	***EUP***	***EUS***	***TS***
**4**	λ, 809	>99	10	14	28	λ, 2682	>99	7	12	79
	λ, 674	>99	12	17	47	λ, 1258	>99	14	19	62
	λ, 781	>99	10	15	36	λ, 1424	>99	8	12	52
	κ, 560	>99	8	11	15	κ, 427	>99	6	9	11
	κ, 782	>99	9	13	27	κ, 603	>99	5	9	20
	κ, 856	>99	8	12	25	κ, 587	>99	5	9	17
**5**	λ, 882	>99	12	15	30	λ, 1793	>99	11	17	67
	λ, 921	>99	15	21	47	λ, 939	>99	12	19	54
	λ, 669	>99	9	14	43	λ, 972	>99	10	15	48
	TTR, 481	>99	9	11	11	TTR, 359	>99	8	9	9
	TTR, 401	>99	7	9	12	TTR, 402	>99	6	8	10
	TTR, 470	>99	7	11	16	TTR, 284	>99	6	8	8
**6**	λ, 350	>99	4	6	10	λ, 513	>99	9	12	26
	λ, 196	>99	5	6	8	λ, 309	>99	5	7	17
	λ, 117	>99	6	8	10	λ, 224	>99	5	5	12
	κ, 60	>99	2	2	3	κ, 0	ND	0	0	0
	κ, 58	>99	2	2	2	κ, 0	ND	0	0	0
	κ, 0	>99	0	0	0	κ, 0	ND	0	0	0
**7**	λ, 287	>99	5	6	14	λ, 997	>99	5	9	29
	λ, 282	>99	6	7	12	λ, 413	>99	6	8	21
	λ, 334	>99	5	5	14	λ, 376	>99	11	12	24
	TTR, 132	>99	2	2	3	TTR, 132	ND	0	0	0
	TTR, 30	ND	0	0	0	TTR, 60	>99	2	2	2
	TTR, 0	ND	0	0	0	TTR, 35	>99	2	2	2

**8**	λ, 553	>99	4	7	26	λ, 914	>99	7	10	34
	λ, 461	>99	4	6	18	λ, 484	>99	3	5	15
	λ, 382	>99	4	5	14	λ, 368	>99	4	7	17
	κ, 119	>99	4	4	6	κ, 0	ND	0	0	0
	κ, 354	>99	5	7	9	κ, 0	ND	0	0	0
	κ, 284	>99	4	5	8	κ, 0	ND	0	0	0
**9**	TTR, 578	>99	7	7	13	TTR, 1799	>99	15	26	63
	TTR, 559	>99	8	9	13	TTR, 1180	>99	13	22	34
	TTR, 619	>99	7	9	11	TTR, 2295	>99	17	27	66
	κ, 680	>99	5	8	20	κ, 277	>99	3	3	9
	κ, 376	>99	5	6	9	κ, 123	>99	4	4	6
	κ, 770	>99	5	7	16	κ, 291	>99	3	3	7
**10**	TTR, 764	>99	15	22	24	TTR, 377	>99	12	16	16
	TTR, 549	>99	10	16	21	TTR, 98	>99	4	4	6
	TTR, 767	>99	12	17	24	TTR, 234	>99	6	8	15
	κ, 296	>99	4	6	8	κ, 66	>99	2	2	2
	κ, 303	>99	4	6	11	κ, 34	ND	0	0	0
	κ, 171	>99	4	6	10	κ, 0	ND	0	0	0

Three technical replicates shown for untreated and decellularised tissue biopsies except for untreated samples for patient 1 and 2 due to lack of material. Proteins identified in each sample with Mascot score probabilities and Scaffold software results: P: Protein identification probability; EUP: Exclusive Unique Peptide count (number of unique peptides only with this protein); EUS: Exclusive Unique Spectrum count (number of unique spectra only associated with this protein); TS: Total Spectrum count (number of total spectra associated with this protein including those shared with other proteins). ND; not detected.

Supplementary Table 2. **MS identification of amyloid signature proteins in cardiac and fat tissue specimen.**

Patient	***Untreated***					***Decellularised***				
	ApoA4	ApoE	SAMP	CLU	VTNC	ApoA4	ApoE	SAMP	CLU	VTNC
**1**		218	86	62	147		51		88	76
**2**	123	233		64			62	103	103	35
**3**	273	115	223	110	302	186	108	335	241	375
**4**	1314	1161	276	260	304	1324	1388	209	428	467
**5**	1612	1386	404	578	1408	737	874	215	427	1622
**6**	321	368	53	169	92	59	315	113	242	200
**7**	551	986	337	234	372	282	891	301	335	477
**8**	716	864	251	219	495	389	1214	297	265	711
**9**	447	293	442	133	331	1006	342	1457	514	853
**10**	478	442	484	275	143	195	167	406	171	89

Identification of proteins associated with the presence of amyloid [1, 2] are shown with Mascot score probabilities (average of three technical replicates except for untreated samples from patients 1 and 2).

Supplementary Table3. MaxQuant quantification of the area of the identified peptides (LFQ) in fat aspirate specimen.

Patient	***Protein***	***Untreated***	***Decellularised***
		LFQ intensity	LFQ intensity
	λ	3492900000	4010100000
	λ	3713800000	3124900000
**4**	λ	3254400000	2729600000
	κ	1205800000	224700000
	κ	1343000000	322380000
	κ	1663700000	299510000
	λ	3568200000	3073900000
	λ	2793200000	3531600000
**5**	λ	2228500000	2650500000
	TTR	197700000	183280000
	TTR	150340000	148220000
	TTR	126730000	139560000
	λ	305010000	268290000
	λ	335660000	272560000
**6**	λ	281080000	250540000
	κ	43558000	0
	κ	45316000	0
	κ	54000000	0
	λ	647330000	104950000
	λ	942160000	94439000
**7**	λ	857270000	53109000
	TTR	0	0
	TTR	0	0
	TTR	0	0
	λ	237050000	69546000
	λ	273100000	54688000
**8**	λ	178810000	48899000
	κ	56321000	0
	κ	86285000	0
	κ	83693000	0
	TTR	292170000	2938000000
	TTR	363270000	3820500000
**9**	TTR	439300000	4053300000
	κ	270020000	28283000
	κ	261410000	38332000
	κ	259610000	37839000
	TTR	863980000	11603000
	TTR	905900000	5365700
**10**	TTR	623670000	12154000
	κ	35173000	212450
	κ	36192000	209520
	κ	71379000	0

Label free quantification (LFQ) intensity values by MaxQuant software (see Methods) are given for each identified protein in three technical replicates per sample. Means of LFQ intensity were used to calculate ratios of soluble versus fibrillar protein before and after decellularisation (see Figure 2B).

Supplementary Table 4. Retrospective clinical and pathologic review of the cases presented in the manuscript.

Patient	Gender	Age (y)	Clinical Diagnosis	Organ involvement	Genetic sequencing
**1**	M	73	AL	Cardiac, soft tissue	
**2**	M	65	AL	Peripheral and autonomic neuropathy	
**3**	M	67	ATTR	Cardiac, peripheral and autonomic neuropathy	TTR T60A
**4**	F	77	AL	Cardiac	
**5**	M	53	AL	Cardiac, soft tissue	
**6**	M	60	AL	Cardiac, renal, liver and autonomic neuropathy	
**7**	F	50	AL	Cardiac, renal and liver	
**8**	F	54	AL	Cardiac, renal and soft tissue	
**9**	M	83	ATTR	Cardiac	Wild type TTR
**10**	M	60	ATTR	Cardiac, peripheral and autonomic neuropathy	TTR A97S

Supplementary references

[1] A. Dogan, Amyloidosis: Insights from Proteomics, Annu. Rev. Pathol. 12 (2016).

[2] P. Mollee, S. Boros, D. Loo, J.E. Ruelcke, V.A. Lakis, K.L. Cao, P. Renaut, M.M. Hill, Implementation and evaluation of amyloidosis subtyping by laser-capture microdissection and tandem mass spectrometry, Clin. Proteomics 13 (2016) 30.
